# Exploring the potential implementation of a tool to enhance shared decision making (SDM) in mental health services in the United Kingdom: a qualitative exploration of the views of service users, carers and professionals

**DOI:** 10.1186/s13033-017-0149-z

**Published:** 2017-06-28

**Authors:** Helen Brooks, Kamelia Harris, Penny Bee, Karina Lovell, Anne Rogers, Richard Drake

**Affiliations:** 1 Mental Health Research Group, Division of Nursing, Midwifery and Social Work, School of Health Sciences, Faculty of Biology, Medicine and Health, University of Manchester, Manchester Academic Health Science Centre, Manchester, M13 9PL UK; 2 Division of Psychology and Mental Health, School of Health Sciences, Faculty of Biology, Medicine and Health, University of Manchester, Oxford Road, Manchester, M13 9PL UK; 3 NIHR CLAHRC Wessex, Faculty of Health Sciences, University of Southampton, Southampton, UK; 4 Institute of Brain, Behaviour and Mental Health, University of Manchester, Room 3.315 Jean McFarlane Building, Oxford Road, Manchester, M13 9PL UK

**Keywords:** Shared decision-making, Collaborative prescribing, Antipsychotic prescribing, Mental health, Qualitative study, Implementation

## Abstract

**Background:**

As a response to evidence that mental health service users and carers expect greater involvement in decisions about antipsychotic medication choice and prescribing, shared decision-making (SDM) has increasingly come to be viewed as an essential element of person-centred care and practice. However, this aspiration has yet to be realised in practice, as service users and carers continue to feel alienated from healthcare services. Existing understanding of the factors affecting the use of tools to support SDM is limited to inter-individual influences and wider factors affecting potential implementation are underexplored.

**Aim:**

To explore the potential use of a tool designed to enhance collaborative antipsychotic prescribing from the perspectives of secondary care mental health service users, carers and professionals.

**Methods:**

We conducted a qualitative study (semi-structured interviews and focus groups) using a convenience sample of 33 participants (10 mental health service users, 10 carers and 13 professionals) involved in antipsychotic prescribing in one Trust in the North of England. Participants were asked about the potential implementation of a tool to support SDM within secondary mental health services. Framework analysis incorporating the use of constant comparative method was used to analyse the data.

**Results:**

The study identified a divergence in the views of service users and professionals, including a previously undocumented tendency for stakeholder groups to blame each other for potential implementation failure. This dissonance was shaped by meso and macro level influences relating to paternalism, legislative frameworks, accountability and lack of resources. Participants did not identify any macro level (policy or structural) facilitators to the use of the tool highlighting the negative impact of mental health contexts. Our study indicated that inter-individual factors are likely to be most important to implementation, given their potential to transcend meso and macro level barriers.

**Conclusions:**

Consideration of the meso and macro level influences identified areas for potential intervention, including challenging professionals’ and service users’ perceptions of each other, rebalancing the notion of accountability within services and introducing new means for service user feedback on the quality of SDM. Multi-level strategies for facilitating the implementation of tools to support SDM are also presented.

## Background

Derived from person-centred approaches to care, shared decision-making (SDM) is a model, that advocates meaningful interaction between professionals and service users to make healthcare decisions that reflect service users’ needs [1–3]. Its purpose is to shift power within health services from professionals to those who use services [4], and aid service users, frontline clinicians, and policy makers in making informed decisions that will improve care quality at individual and population levels. SDM has been shown to be a priority for service users, and has improved treatment adherence and satisfaction in various conditions [5]. SDM is seen as emphasising respect for individual agency, autonomy and an emphatic approach to practice [6].

Traditionally, SDM has been conceptualised as involving four key components: (i) the involvement of at least two parties (e.g. the service user and professional), who (ii) share information, (iii) attempt to reach consensus and (iv) reach a decision [7]. More recently, the notion of ‘collaborative deliberation’ [6] as a theoretical model underpinning SDM has been suggested. Collaborative deliberation upholds that SDM is a dynamic process and comprises of a set of important elements: constructive interpersonal engagement, recognition of alternative actions, comparative learning, and preference construction elicitation and integration.

Despite its theoretical underpinnings, SDM has proved challenging to operationalise in practice, not least because it implies, by definition, the undertaking of significant interpersonal relational work [8]. Acknowledged barriers from the staff perceptive include lack of time and motivation, engrained professional identities and the complexities of a working in a multidisciplinary setting that can limit trust and communication between different specialities and sources of experience [9]. A recent Cochrane Systematic review has demonstrated that the quality of evidence concerning the effectiveness of SDM is low [10].

Antipsychotic prescribing in mental health care presents a unique opportunity for the enactment and evaluation of SDM. At a most basic level, anti-psychotic prescribing usually involves a choice between two or more similarly effective drugs, each with differing adverse effects. However, research reveals a more complex relationship operating, with multiple social influences impacting on the ‘choice’ to take anti-psychotic medication. Such influences include the view of significant others, service users’ relationships with prescribers, and service user sand professional concerns about the risk of coercion [11, 12]. Effective SDM in the context of medication prescribing thus requires a wider understanding of the role of social networks (e.g. the role of significant others in a service user’s life) as well as the symbolic significance attached to antipsychotic medication by those that take them. Examples from the literature that can discourage pharmacological use include the role of medication as a vehicle of social control [12], potential resistance to taking medication because of side-effects [13], and the stigma that might ensue from being identified as someone taking anti-psychotic medication [12].

Service users, carers and professionals express support for active involvement in decisions about the prescribing of medication but there is evidence that they do not feel adequately involved in this process [14–16].

### Decision tools and how they contribute to SDM

Existing evidence of the effects of decision support aids on SDM processes and outcomes is mixed [17, 18]. Identified benefits include process-related or short-term outcomes related to the acknowledgement of service user preferences, attitudes and behaviours [17]. Longer-term effects are uncertain [18].

The ways in which mental health innovations are implemented in practice, and their relative success in being embedded and sustained, have become recent and popular foci for research [19–21]. Translational gaps are widely acknowledged, but implementation processes in mental health services are still poorly understood [22].

Nilsen outlines three approaches used in the implementation of research into routine practice: (i) determinant frameworks, classic theories and implementation theories which help understand or explain what influences implementation outcomes; (ii) process models which describe/guide the process of translating research into practice; and (iii) evaluation frameworks which evaluate implementation [23]. Determinant frameworks can be too generic and provide limited support for context-sensitive implementation [21]. Process models do acknowledge the importance of addressing barriers and enablers to the translation of research into practice, but rarely identify, or systematically refer to and structure, specific determinants of successful mental health implementation. It is this gap, which the current study sought to address [23].

Potential barriers to the implementation of a SDM tool for anti-psychotic prescribing are identifiable from existing research [24–32]. They include service users’ characteristics including cognitive skills, articulacy and level of illness, a lack of shared definition of ‘involvement’ between service users and mental health professionals, disparity in information and power in the clinical situation, and professional concerns about service users’ capacity and time pressures [24–32]. Key enablers include clinician motivation, and the practicality and consistency of SDM use in the clinical context [24–32]. However, the bulk of existing research has focussed on micro social barriers to implementation, with little consideration given to the meso (health care organisation level) or macro (structural level) influences on the implementation of interventions designed to enhance shared decision making. Obtaining a more complete picture prior to implementation of the potential facilitators and barriers to implementation of such solutions, including those at a higher contextual level, is likely to be necessary to ensure translation and embedding into mental health services [20]. The fundamental principle underlying the use of a macro-meso-micro framework is that to understand the impact of service innovations, the interconnection between meanings across these multiple organisational levels is required [33]. Working in and across these multiple levels develops understanding of factors related to the successful implementation of tools to support the SDM process and also identifies where and why this may not occur [33].

This study extends previous qualitative research of the values and use of SDM by exploring the views of multiple stakeholders (service users, carers and psychiatrists) within one Mental Health Trust on the potential use of a decision making tool (described later) to aid collaborative prescribing. It will also consider specific determinants of successful implementation to address the aforementioned criticisms of other process models of implementation [23]. Three different levels of implementation (micro, meso and macro) were used as a thematic framework to guide the holistic design and analysis of the study advocated to consider the context and social norms in which tools are enacted [11].

## Methods

### SDM tool

This paper-based tool, developed by clinical academics from the University of Manchester to support SDM within mental health services was designed to prompt individual engagement by encouraging service users (and their carers where appropriate and desirable) to consider the five side effects they would most wish to avoid when taking antipsychotic medication, to which the professional matches varieties of medication which meet these requirements (Appendix 1). These are then recorded on the tool, which prompts and facilitates empathetic discussion between service users, carers and professionals about the most appropriate medication to prescribe for the individual concerned. Information cards detailing information on additional side effects related to the matched medication choices are also shared with service users and carers during this process to support collaborative discussion and enable autonomous decision making (example included in Appendix 2). Current evidence as to the effectiveness of tools to support SDM in mental health settings is inconclusive [17, 18]; however, It has been demonstrated that interventions designed to promote SDM targeting multiple stakeholder groups show more promise than those only targeting one or the other [10].

#### Study design

We conducted a qualitative study incorporating focus groups and one-to-one interviews with mental health service users, carers and professionals within one Mental Health Trust in the UK. To be eligible to take part in the study, participants had to be over 18 years old and have had some experience of antipsychotic prescribing as a service user, as a friend or family member who cared for someone prescribed antipsychotic medication, or as a professional at the relevant NHS Trust. Posters advertising the study were displayed in Trust premises. Participants who were interested in taking part in the study contacted the research team directly by phone or email for further information. Those who could not, or did not want, to attend a focus group were offered a one-to-one interview as an alternative. The study was approved by NRES Committee North West-Lancaster (REC Reference: 15/NW/0070).

### Sample

Our convenience sample comprised 19 service users and carers, and 13 mental health care professionals. Of the 10 service user participants, two also identified as a carer and five were female. The majority of carer participants were female (90%, n = 9). The majority of professional participants were Consultant Psychiatrists (77%, n = 10) but also included Community Psychiatric Nurses (15%, n = 2) and one Pharmacist (8%). See Table 1 for further details on study participants. Recruitment stopped when the research team agreed during analysis meetings that saturation of data had occurred and were confident no new themes were arising from the data.

**Table 1:** Demographic information

Service users (2 identified as carers too)	
Male	5
Female	5
Total	10
Carers	
Male	1
Female	9
Total	10
Professionals	
Male	10
Female	3
Consultant Psychiatrists	10
Community Psychiatric Nurses	2
Pharmacist	1
Total	13

#### Data collection and analysis

Interviews and focus groups took place from April 2015 to October 2015 (see Appendix 3 for a summary topic guide which was informed by existing literature including the previously identified facilitators and barriers to the implementation of tools to SDM [24–32]). Interviews and focus groups were led by HB and KH who are experienced qualitative researchers with backgrounds in mental health and health services research. The focus of the interviews and focus groups was to elicit views and experiences of antipsychotic prescribing and explore the potential multi-level implementation of the proposed tool to support collaborative prescribing within health services.

Informed consent was obtained by researchers before the focus groups and interviews were conducted. In those instances whereby professional interviews were carried out over the phone (n = 5), consent forms were posted out, completed and returned prior to data collection taking place. Interviews conducted face-to-face and focus groups lasted approximately one hour whereas telephone interviews lasted between 20 and 45 min.

We carried out five focus groups (four with a mixture of service users and carers at participants' request and one with professionals). In addition to these focus groups, we conducted eight face-to-face interviews (two with service users, one with a carer and five with professionals) and five telephone interviews (all professionals). Focus groups and interviews were undertaken either on University or Trust premises. Once the focus groups and interviews were complete, the audio files were downloaded from the encrypted audio-recorder and transcribed verbatim. Upon receipt, transcripts were anonymised.

Transcripts were analysed using Framework Analysis, which allows for the use of both inductive and deductive coding [34]. The analysis team involved academic researchers working alongside a service user-researcher for purposes of independent coding. All those involved in the analysis read the transcripts allocated to them on numerous occasions in order for them to become fully immersed in the data and ensure familiarity. Data were managed using NVivo 10, along with a word document containing emerging themes, to provide a data trail. Four of the authors (HB, KH, PB and RD) met regularly to discuss emergent themes and negative cases. HB and KH developed a coding framework by independently coding the first four transcripts, which was then used to apply codes to the remaining data (3 main codes with 61 sub-codes). PB and RD conducted double coding of four transcripts to ensure rigour and the trustworthiness of analysis undertaken (eight transcripts in total were coded independently by at least two members of the research team), as well as to discuss redundant or overlapping codes. A service user-researcher coded two transcripts against the original framework to ensure codes were grounded in the data. To further ensure confidence for the reader in relation to the trustworthiness of data, we presented our framework to an advisory group from a related project made up of 12 mental health service users and carers in February 2016. The advisory group fed back on the themes which allowed for further refinement of the framework. This iterative process allowed for constant comparison and the development of a final framework (3 main codes with 36 sub-codes) which was considered to best represent the data provided during interviews and focus groups.

HB, KH and PB are mental health service researchers, KL is a Professor in Mental Health, AR is a Professor of Health Systems Implementation and RD is a Consultant Psychiatrist. The research team had no therapeutic relationship with the service users or carers included in the study. RD worked in the same Trust as some professional participants. All data were collected by HB and KH and only shared with other members of the research team in an anonymised format. The study team was made up of a mixture of those working within and outside of mental health services to balance any pre-formed views about the nature of prescribing from the literature or practice.

## Results

All stakeholders agreed that there was a need for current SDM practices to be improved and perspectives of current process are reported elsewhere [35]. Participants felt unanimously that there was potential value to the proposed tool but identified a range of micro, meso and macro level barriers and facilitators to its implementation (Table 2). By every demographic measure the professional group differed from other groups more than the carers and users differed in terms of the implementation factors they identified. There were no discernible differences in the data collected via focus groups or individual face-to-face interviews or within stakeholder groups.

**Table 2:** Summary table of multi-level positive and negative influences on the implementation of the SDM tool based on qualitative data along with strategies to promote the use of SDM at each level

Level	Positive influences on potential implementation	Negative influences on potential implementation	Strategies to promote the use of SDM tool within services
Macro level (structural)		Mental Health Act	Introduce additional accountability mechanisms related to the quality of SDM and associated outcomes
		Protocol driven practice	Introduce quality targets associated with the quality of SDM and use of the tool and promote within services
		Resource limitations	Seek and act on the feedback from of a range of stakeholders (service users, carers and professionals) about the tool and wider SDM within services and how this might be improved
		Culture	
		IT systems	
Meso level (healthcare)	Community setting	Culture (risk focussed, paternalism)	Provide holistic care
	AOT approach	Access to professionals	Provide recovery focussed care
	Information provision	Lack of medication choice	Provide information about the tool, how it works, available options and what users are entitled to
	Activity provision	Lack of information sharing	Provide wider engagement activities within the Trust
	Holistic approach		Provide, promote and utilise mechanisms for service users and carers to feedback about the quality of the tool, SDM and associated outcomes
Micro level influences			
Service user/carer	Insight	Lack of insight	
	New to services	Behaviour (delusions, paranoia)	
	Role of carer	Crisis/lack of capacity	
		Perception of services	
		Institutionalisation	
Professional	Behaviour (compassion, conviction)	Focus on psychosis	Actively involve carers in the use of the tool
	Relationships	Limited contact	Consider carer involvement in situations (acute illness) where service user involvement is not possible
	Communication	Offering choice looking like uncertainty	Make explicit the purpose and boundaries of SDM within a given situation when using the tool
		Authority and power	Highlight varieties and boundaries of SDM possible within different contexts when using the tool (e.g. choice of medication for those detained under the Mental Health Act)
			Treat the use of the tool as an on-going process which is revisited continually

### Micro level factors—insight, honesty and communication: the role of interpersonal relationships within health services

The successful implementation of the proposed SDM tool was considered dependent on adequate relationships between service users, carers and professionals that were underpinned by a number of service user and professional characteristics.

### Service user/carer characteristics

Most micro level barriers to implementation were specific to severe mental illness, rather than arising from features general to all health care. There was a divergence in views about barriers to the implementation of the tool within health services. Professionals, in particular, focussed on the negative attributes of service user characteristics and how these might impact on potential implementation. The most prominent barrier reported by professionals was a ‘lack of insight’ on the part of service users to enable them to engage in discussions about medications and side effects.

*If they’ve not got insight and they don’t believe they’re mentally ill and I’ll say, well why are you taking your tablet and they’ll just say, cos the doctor tells me to.* ***Professional Interview ID4: Community Psychiatric Nurse***

‘Insight’ was considered a prerequisite to the use of the tool and SDM more generally and professionals acknowledged that insight fluctuated over time. At moments when professional considered service users to be demonstrating florid symptoms, insight was considered to be lacking. In these situations, professionals felt that they needed to make decisions for service users and that use of the tool would not be appropriate.

*Some clients we have, have fluctuating insight or… poor insight and then we have to take a more sort of paternal role with their medication really.* ***Professional Interview ID5, Community Psychiatric Nurse.***

Participants raised other facets of service users’ capacity to engage with the tool that were potential micro level barriers to implementation. These included acute periods of illness during which service users may be unable, or lack the motivation, to engage in healthcare discussions, specific behaviours such as delusional and paranoid behaviour, as well as a general rejection of health services and medication generally.

*Sometimes there will be people [that] are anti*-*medication…that’s not just a mental health thing, you, you get that everywhere…* ***Professional interview ID1, Pharmacist.***

Carers felt in these situations, they could play a vital role advocating on behalf of a service user in discussions about side effects and choice of medication given their in-depth knowledge of the person they cared for. Professionals, however, were more likely to describe the role of carers in terms of advocating a decision to the service user.

*But in the few clients we’ve had whose family have been involved that’s been a help, if you can sort of kind of get the family onside to the treatment and if the family can recognise the need for treatment and the importance of it…that can help.* ***Professional Interview ID5, Community Psychiatric Nurse***

Participants raised individuals’ past relationships with health services as a potential factor that may impact on the use of the SDM tool. Whilst the optimal candidate for use would be a service user perceived to be stable by services (given the aforementioned concerns about capacity), participants felt that someone who was new to services may be more likely to engage with the tool. This was because they would not have become ‘institutionalised’ by services nor would they have experienced failed treatment previously.

*Because you’ve got more…perhaps more scope, in terms of… they’ve not had previous treatment failures.* ***Professional interview ID2, Consultant Psychiatrist***.

### Professional characteristics

Service users, carers and professionals talked about successful use of the tool requiring professionals who displayed certain behavioural practices. These characteristics included taking the time get to know the service user, being compassionate, not focussing on psychosis alone, having adequate knowledge and being able to convey this successfully, as well as having conviction in the decisions that were made. These attributes facilitated communication with service users, which contributed directly to dyadic relationships. The crucial component of these relationships from the service users’ point of view related to trust in the professional treating them and feeling that their voices were being heard. This allowed for open and honest dialogue between relevant stakeholders and was considered a cornerstone of the successful use of the tool. Unsurprisingly, these relationships were perceived to be facilitated by continuity of care. There was an acknowledgement that the proposed tool may contribute to these required components of SDM by providing a vehicle to open up conversations and discussions.

*You want to have the discussion with the patient but you need to have everything ready and know how you’re going to present it to the patient in a way that they will be able to understand and weigh things up…and also patients are more likely I think to trust you and to take part in that decision and feel like it’s a worthwhile decision to take part in if they feel that you have done your homework on it as it were.* ***Professional interview ID 1, Pharmacist***.

This open and honest dialogue was hindered by misconceptions on both sides of the health service relationship. For example, service users felt that professionals may not be open to or fully trust their opinions or may not be transparent about medication side effects because they did not want them to stop taking their medication. Similarly, professionals felt that service users might not be honest about their experience of medication, as they did not want to take medication.

*And in fact if you, if you refuse it… they sort of like look at you as if to say we don’t believe you’re ill like you say you are.* ***Service User/Carer Focus Group 3.***

Additionally, some professionals felt that by offering the choice required by the proposed SDM tool, this would reflect uncertainty on their part and detract from their professional expertise and authority. Service users did not universally value this notion of authority and reported that it was difficult to raise issues of concern in consultations if these were not initiated by the professional because of a perceived power differential. However, service users and carers also acknowledged that there were times (e.g. in times of crisis or periods of acute illness) when they would wish to defer to the professional’s opinion.

### Meso level (organisational)—the role of holistic approaches to service provision and importance of adequate time and resources

There were various meso level factors affecting the potential implementation of the SDM tool reported by participants included in the study. Facilitators included healthcare organisations that utilised a holistic approach (gave consideration to all aspects considered important to service users) to treatment and provided recovery-based activities to engage service users more generally with services. These included community-based teams and those utilising an assertive outreach approach, in particular. These were compared to other settings, such as inpatient settings, which were considered to have cultures with a greater focus on risk, which would render it more difficult to have the type of dialogue advocated by the tool. It may be that these types of settings are also less constricted by the coercive and legislative frameworks to be discussed in the next section.

*When Assertive Outreach Treatment (AOT) first started, they very much used to take an [holistic] approach… When I did some work for them as a trainee, very much, okay, you don’t want to talk about your psychosis, let’s talk about where you live or your bills or your debt.* ***Professional focus group 1.***

Information provision was another key facilitator to potential implementation reported by stakeholders. Information about available medication choices needed to be shared with participants in an easily digestible and understandable format, giving details about how and why the medication worked, the likelihood of anticipated side effects and any relevant interaction effects. This information would need to be updated as new evidence became available and provided to service users and carers in a suitable way to enable them to make informed decisions in collaboration with their professional when using the tool.

*I also think you need to have almost like a medication review, because if there is a new medication out there where there are less side effects or you can be on a lower dose and still have the same, you know, just making use of research, so that we know that you don’t have to stay at twenty milligrams.* ***Service user and carer focus group 1.***

The main barrier to the use of the tool described by participants at the meso level was that of limited human resources within their healthcare organisation, which hindered the time professionals had to spend with service users and carers.

*I’ve got half an hour per patient in the ward round, but that includes talking to the nursing staff, the junior doctor, the care coordinator, members of the family, any… anybody else and then the patient gets to come in as well at some point in that half an hour.* ***Professional focus group 1***

Time was considered critical to successfully implement and use the tool within health services and this was something that all stakeholders felt was missing from current healthcare climates due to increased work and caseloads. This also limited access to psychiatrists, alternative treatments and the availability of different types of medication within services. Without the required resources, participants felt there was a pressure to prescribe medication quickly within services, which was a significant barrier to the use of the proposed tool.

*I know that the Trust would probably would want me to slam them on something straight away and let’s get on with it.* ***Professional focus group 1***

### Macro (policy and structural) factors—the competing agendas of SDM and legislative contexts within mental health services

There were no macro level facilitators to the use of the tool as reported by participants included in this study. It appeared that the reason for this was that the values enshrined in SDM and the tool itself assume the sharing of responsibility for decisions between the relevant parties but these values are diametrically opposed to the coercive and legislative frameworks within wider mental health contexts.

*You’d get clients hiding medication in their mouth or under their tongue or even vomiting after medication to try and get rid of it so… quite problematic and, [it], often ended up in a depot medication or some other form of sort of enforced medication like tablets that melt on the tongue or things like that.* ***Professional, focus group 1***

Participants included in this study did not appear to consider that some level of involvement with the tool could still be negotiated in these circumstances. In fact, a small number of service users went further. They pointed out that the threat of being detained against their will under the Mental Health Act was occasionally used as a coercive strategy to encourage compliance with medication and, as a result,  implied that even when service users were not detained, meaningful collaborative choice through use of the tool might not be possible. Service users made the point that when their rationality was questioned, even if only indirectly by consideration of the use of the Mental Health Act, this indicated to them that clinicians did not consider their preferences valid. They implied that this validation was a prerequisite for meaningful involvement with the SDM tool.

*So by me telling you that those tablets that you’ve given me aren’t suitable for me, you’re telling me I’m going to section you because I won’t take the tablets.* ***Service user interview ID1.***

Resource limitations at the macro level are likely to impact on the reports of limited resources at the meso healthcare organisation level, given the top-down structure of mental health services. Top-down protocol-driven practice (including the risk assessment process) was also considered a structural barrier to the nuanced negotiations required to successfully use the tool because they reduced flexibility in terms of the options available for professionals and service users. For the SDM tool to be successfully implemented, participants felt it should not become another bureaucratic ‘tick box’ exercise or take away from face-to-face consultations, and, instead represent a way to prompt SDM discussions and open up on-going discussions about choice with service users and carers.

*Sometimes we are, [] losing common sense and just because something is not written somewhere or something is not done because of that protocol driven, kind of, approach is a bit of a restriction and the other bit is this thing about we are focussed on discharge now.* ***Professional focus group 1***

## Discussion

This study addresses a gap in the literature on the implementation of tools to support the SDM process within mental health services. Guided by theories of implementation science, it identified several micro, meso and macro level factors from the point of view of service users, carers and professionals in one Mental Health Trust, providing new insights when considering the implementation of tools to support SDM. Our study sought to understand and simplify these facilitators and barriers related to potential implementation and to provide insight into these processes at different levels (Table 2). The majority of barriers reported by participants coalesced at the micro relational level in line with previous studies [11, 14, 24–32]. What this study also adds to existing literature is an in-depth consideration of the meso and macro level factors and, in particular, that participants identified no macro level facilitators to the use of the tool within the wider healthcare context. Table 2 details multi-level strategies, derived through a synthesis of facilitators raised by participants, to enhance the success of the tool support SDM in services.

The analysis identified an important disconnect between service users and professionals not highlighted previously. Stakeholders blamed each other for a potential failure to implement the tool successfully in practice. Professionals thought the implementation of a SDM tool would fail because of negative attributes of service users’ behaviour, and similarly service users were not confident that SDM discussions prompted by the tool would result in demonstrable accountable service-level decisions. The concept of ‘insight’ is a complex and highly subjective term often used to sustain the power imbalance within the mental health system [36]. Despite these criticisms, professionals still cited service user ‘insight’ as the greatest barrier they perceived to the use of the tool, with no acknowledgement in the professional lexicon of a need to, or ways of addressing this. Service efforts should focus on how to involve service users and carers within the decision-making process, even during times of detention or acute illness which may go some way to address the divergence of views identified within this study.

The study results confirm that in different ways all stakeholders are discontented with the current degree of collaboration in prescribing decisions. Service users were sometimes concerned with the possibility of compulsory treatment and some also felt that they could not challenge decisions about prescribing sufficiently. This made some sceptical about the introduction of shared decision making [4, 11]. Considering how service users and carers feedback about the success of SDM interventions might be important as a means of establishing their credibility in the process as well as monitoring success.

Although in mental health services, as in most areas of contemporary healthcare, patient choice is increasingly emphasised, mental health professionals and services have a unique duty to consider whether to override service users’ choices under the terms of the Mental Health and Mental Capacity Acts. With the 2007 amendment of the Mental Health Act, Community Treatment Orders extend this by enabling services in some cases to detain people simply for stopping medication. Moreover service users reported a range of other strategies being used by professionals that some saw as coercive. It is clear that some service users felt profound alienation from services on this basis. However, our study indicates that at the point of intervention, micro level factors might be most powerful, given that meso and macro level barriers to implementation may be overcome when a supportive dyadic relationship is achieved whereby mutually beneficial decisions about medication can be made through the use of the tool, based on honest and open dialogue and mutual trust.

Although participants included in this study mentioned no macro level facilitators to the use of the SDM tool, there are numerous policy and practice mandates in this regard. For example, NHS policy has shifted towards person-centred care [37] and external stakeholder groups such as service user groups, the pharmaceutical industry and various academics encourage SDM [38]. The culture of some mental health services includes the valuation of autonomy and family involvement in a range of circumstances (thus limiting the application of the Mental Health and Mental Capacity Acts) and this is exemplified by the interest that a range of professionals have in the practice of SDM, both here and elsewhere in the literature [2, 3]. Stakeholders identified a range of micro, meso and macro level constraints on SDM that appeared to dominate actual practice as recounted by the participants [24–32]. This implies that in order to realise the full potential of tools such as the one described here, the whole range of these barriers needs to be addressed as far as possible to ensure that all the required elements of the dynamic process of SDM can be realised in practice [6, 24–32]. Also, it may be possible to collaborate further than is implied by the negativity of the discourse here—the fact that participants focus on the barriers suggests a shared dissatisfaction with the status quo and an appetite to be more collaborative that is thwarted by a lack of a clear means to address the situation in practice.

The study results provide new insights on the practical implementation imperatives involved in introducing a tool designed to improve SDM within mental health services. The analysis highlights the critical roles of all stakeholders (professionals, service users and carers) in order for successful implementation to be achieved. It also highlighted the need for the use of such tools to support SDM to be an individualised and on-going process due to the floridity of illness as well as the use of different strategies to interact with service users and carers at different times of illness [29] (Table 2).

This study gains its strengths from the qualitative nature of the focus groups and interviews conducted. Including multiple stakeholders from one NHS Trust allowed for an in-depth exploration of the multi-level facilitators and barriers to implementation, along with a direct comparison of stakeholder views to aid understanding. Utilising a micro-meso-macro implementation framework to guide the study resulted in a more complete understanding of the difficulties facing health service providers when trying to implement tools to support SDM. The contributions of service users, carers and professionals to the analysis process ensured the trustworthiness of data and validity of themes arising from the data. However, participants were all recruited via poster advertisements in Trust premises. It is therefore possible that those included in the study were particularly motivated to take part in research with particular opinions at a certain point in recovery. The majority of professional participants were Consultant Psychiatrists, which is justified on the basis that they tend to be the main prescriber, and are thus most relevant for SDM implementation. The majority of carer respondents were women, which may have impacted on the viewpoints expressed in this study and should be considered when interpreting the results. Service users in different positions may have reported different facilitators and barriers. For example, research has suggested that those from middle class backgrounds are more likely to participate in healthcare decisions than their counterparts [39]. Additionally, those from different ethnicities may have raised different issues [40]. Focus groups involved both service user and carer participants and, whilst this did not appear to be the case, it should be acknowledged that this might have impacted on the level of disclosure from service users and/or carers.

## Conclusions

This study provides a new understanding of the micro, meso and macro level barriers to the implementation of tools to support SDM in secondary care mental health services, which are generalisable to other SDM contexts, such as wider prescribing in mental health services and other treatment related decisions. The study identified dissonance at the individual level between service users and professionals, which was shaped by meso and macro level influences relating to paternalism, legislative frameworks, accountability and lack of resources. These findings have direct relevance to policy and practice through incorporating a consideration of these multi-faceted factors into implementation strategies. Such approaches are more likely to improve care for those with serious mental illness and offer a chance to rebalance strained relationships between staff, service users and carers.

## Appendix

### Appendix 1: Shared-decision making tool for service users to complete

#### Instructions

Pick the 5 side effects that you most want to avoid. Then number them from 1 (most want to avoid) to 5 (least want to avoid). If there are any that would be helpful, tick them (or example, sleepiness at night if you have poor sleep or weight loss if you want to lose weight).

**Table:**

Difficulty staying awake during the day	
Sleeping late in the mornings	
Sleepy in the evening or at night	
Tiredness	
Difficulty getting off to sleep	
Increased dreaming	
Weight gain	
Weight loss	
Feeling sick	
Over-wet or drooling mouth	
Dry mouth	
Constipation	
Risk of high blood sugar/diabetes	
Risk of high cholesterol	
Less sex drive	
Difficulty getting an erection	
Difficulty having an orgasm	
Periods stopping	
Periods irregular and not often	
Increased sweating	
Difficulty passing urine	
Blurred vision	
Difficulty remembering things	
Tension	
Slowing of movements	
Stiff muscles	
Muscle spasms	
Restlessness	
Parts of the body restless, seem to move by themselves (e.g. feet)	
Small risk of abnormal movements	
Shaking or tremor	
Dizziness on standing up	
Higher blood pressure	
Sensitivity to sun	
Noticing your heart beating fast (palpitations)	
Small risk of heart problems	

Reproduced with permission from the authors at the University of Manchester

### Appendix 2: Example of information card given to service users during use of the tool

#### Aripiprazole

- Poor sleep++
- Restlessness ++
- Feeling sick, not hungry +

+ uncommon or mild; ++ commoner or moderately severe; +++ common or more severe.

Reproduced with permission from authors from the University of Manchester.

## Abbreviations

AOT: assertive outreach team
SDM: shared decision making
US: United States
